# Amino­guanidinium hydrogen succinate

**DOI:** 10.1107/S1600536809003626

**Published:** 2009-02-04

**Authors:** S. Murugavel, P. S. Kannan, A. Subbiah Pandi, S. Govindarajan, R. Selvakumar

**Affiliations:** a Department of Physics, Thanthai Periyar Government Institute of Technology, Vellore 632 002, India; b Department of Physics, S. M. K. Fomra Institute of Technology, Thaiyur, Chennai 603 103, India; c Department of Physics, Presidency College (Autonomous), Chennai 600 005, India; d Department of Chemistry, Bharathiar University, Coimbatore 641 046, India

## Abstract

The title compound, CH_7_N_4_^+^·C_4_H_5_O_4_^−^, is a molecular salt containing discrete amino­guanidinium and succinate ions. The amino­guanidinium cation is nearly planar, with a maximum deviation of 0.035 (1) Å. The dihedral angle between the amino­guanidinium cation and the succinate anion is 3.35 (6)°. The crystal packing exhibits inter­molecular N—H⋯O and O—H⋯·O hydrogen bonds.

## Related literature

For related structures, see: Adams (1977 ▶); Mullen & Hellner (1978 ▶); Akella & Keszler (1994 ▶). For biological applications of amino­guanadine, see: Makita *et al.* (1995 ▶); Brownlee *et al.* (1986 ▶). For graph-set notation, see: Bernstein *et al.* (1995 ▶). 
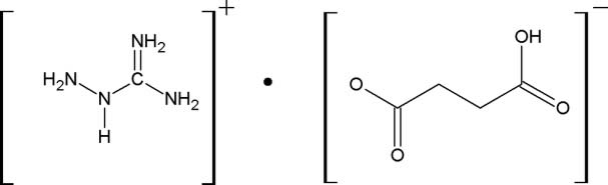


## Experimental

### 

#### Crystal data

CH_7_N_4_^+^·C_4_H_5_O_4_^−^
*M* *_r_* = 192.19
Monoclinic, 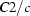

*a* = 15.071 (5) Å
*b* = 6.565 (2) Å
*c* = 18.152 (5) Å
β = 109.733 (5)°
*V* = 1690.5 (9) Å^3^
*Z* = 8
Mo *K*α radiation
μ = 0.13 mm^−1^
*T* = 293 (2) K
0.25 × 0.16 × 0.16 mm

#### Data collection

Bruker APEXII CCD diffractometer
Absorption correction: multi-scan (*SADABS*; Sheldrick, 1996 ▶) *T*_min_ = 0.968, *T*_max_ = 0.980
11302 measured reflections
2773 independent reflections
2107 reflections with *I* > 2σ(*I*)
*R*_int_ = 0.021

#### Refinement

*R*[*F* ^2^ > 2σ(*F* ^2^)] = 0.043
*wR*(*F* ^2^) = 0.135
*S* = 1.05
2773 reflections
146 parameters
H atoms treated by a mixture of independent and constrained refinement
Δρ_max_ = 0.37 e Å^−3^
Δρ_min_ = −0.27 e Å^−3^

### 

Data collection: *APEX2* (Bruker, 2004 ▶); cell refinement: *SAINT* (Bruker, 2004 ▶); data reduction: *SAINT*; program(s) used to solve structure: *SHELXS97* (Sheldrick, 2008 ▶); program(s) used to refine structure: *SHELXL97* (Sheldrick, 2008 ▶); molecular graphics: *ORTEP-3* (Farrugia, 1997 ▶); software used to prepare material for publication: *SHELXL97* and *PLATON* (Spek, 2003 ▶).

## Supplementary Material

Crystal structure: contains datablocks global, I. DOI: 10.1107/S1600536809003626/lx2087sup1.cif

Structure factors: contains datablocks I. DOI: 10.1107/S1600536809003626/lx2087Isup2.hkl

Additional supplementary materials:  crystallographic information; 3D view; checkCIF report

## Figures and Tables

**Table 1:** Hydrogen-bond geometry (Å, °)

*D*—H⋯*A*	*D*—H	H⋯*A*	*D*⋯*A*	*D*—H⋯*A*
N10—H10*B*⋯O9	0.87 (2)	1.99 (2)	2.851 (1)	171 (2)
N11—H11⋯O8	0.88 (2)	2.07 (2)	2.939 (1)	166 (1)
N12—H12*B*⋯O6^i^	0.85 (2)	2.07 (2)	2.921 (1)	178 (2)
N10—H10*A*⋯O7^i^	0.84 (2)	2.05 (2)	2.886 (1)	178 (2)
O6—H6⋯O8^ii^	0.82	1.65	2.456 (1)	167

Symmetry codes: (i) 
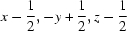
; (ii) 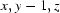.

**Fig. 1.**
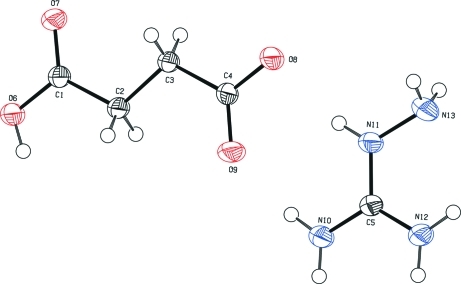
The molecular structure of title compound showing 50% probability displacement ellipsoids.

**Fig. 2.**
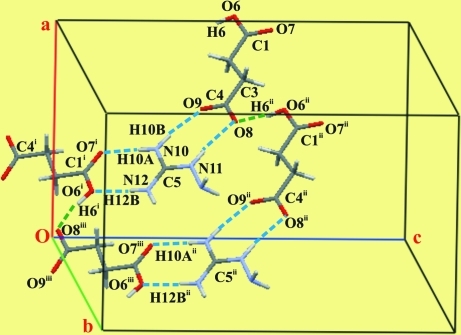
N—H···O and O—H···O hydrogen bonds (dotted lines) in the title compound. [Symmetry code: (i) x - 1/2, -y + 1/2, z - 1/2; (ii) x, y - 1, z].

## supplementary crystallographic information

### Comment

Aminoguanadine is an early inhibitor of Advanced Glycosylation End products (AGEs) (Makita *et al.*, 1995). It helps prevent proteins cross-linking and is being used in diabetes, atherosclerosis, renal and aging disorders (Brownlee *et al.*, 1986). Aminoguanadine is a highly reactive nucleophilic reagent that reacts with many biological molecules (Pyridoxal phosphate, Pyruvate, glucose, malondialdehyde, and others). The crystal structures of several guanidinium salts have previously been reported over the last three decades (Adams, 1977; Mullen & Hellner, 1978). Here We report the crystal structure of the title compound, aminoguanidinium hydrogensuccinate (I) (Fig. 1).

The aminoguanidinium is nearly planar, with atom N11 shows the maximum deviation from planarity 0.035 (1) Å. The bond lengths in (I) are normal and comparable with the corresponding values observed in the related structure (Akella & Keszler, 1994). The dihedral angle between the aminoguanidinium cation and succinate anion is 3.35 (6)°. Two main motifs dominate the hydrogen bond in (I). Firstly, a nearly symmetrical simple *R*_2_^2^(8) ring (Bernstein *et al.*, 1995) forms from hydrogen bond between the two molecules involving the two guanidinium amino groups and the two succinate O atoms, *viz.* N10—H10B···O9 and N11—H11···O8 (Table 1 and Fig. 2). Secondly, atom N12 and N10 in the molecule at (*x*, *y*, *z*) donate one proton each to atom O6 and O7 in the molecule at (-1/2 + *x*, 1/2 - *y*, -1/2 + *z*), generating *R*_2_^2^(8) ring motif (Table 1 and Fig. 2).  Also, the O—H···O interaction is observed (Table 1). Thus, the symmetry-related molecules are cross linked by these hydrogen bonds to generate a three-dimensional network.

### Experimental

Aminoguanidine bicarbonate (0.136 g; 0.001 mol) was added in small portions with stirring to an aqueous solution (30 ml) of succinic acid (0.118 g; 0.001 mol). The resulting clear solution of pH<2 was concentrated over water-bath to half of its volume. The transparent single crystals suitable for X-ray diffraction obtained by slow evaporation at room temperature were separated, washed with ethanol and air dried.

### Refinement

All N bound H atoms were located in a difference map and refined freely. All other H atoms were fixed geometrically and allowed to ride on their parent atoms, with O—H = 0.82 Å and C—H = 0.97 Å with *U*_iso_(H)=

1.2*U*_eq_.

### Crystal data

**Table:**

CH_7_N_4_^+^·C_4_H_5_O_4_^−^	*F*(000) = 816
*M**_r_* = 192.19	*D*_x_ = 1.510 Mg m^−^^3^
Monoclinic, *C*2/*c*	Mo *K*α radiation, λ = 0.71069 Å
Hall symbol: -C 2yc	Cell parameters from 2773 reflections
*a* = 15.071 (5) Å	θ = 2.4–31.4°
*b* = 6.565 (2) Å	µ = 0.13 mm^−^^1^
*c* = 18.152 (5) Å	*T* = 293 K
β = 109.733 (5)°	Block, colourless
*V* = 1690.5 (9) Å^3^	0.25 × 0.16 × 0.16 mm
*Z* = 8	

### Data collection

**Table:**

Bruker APEXII CCD diffractometer	2773 independent reflections
Radiation source: fine-focus sealed tube	2107 reflections with *I* > 2σ(*I*)
graphite	*R*_int_ = 0.021
Detector resolution: 10.0 pixels mm^-1^	θ_max_ = 31.4°, θ_min_ = 2.4°
ω scans	*h* = −22→21
Absorption correction: multi-scan (*SADABS*; Sheldrick, 1996)	*k* = −9→9
*T*_min_ = 0.968, *T*_max_ = 0.980	*l* = −25→26
11302 measured reflections	

### Refinement

**Table:**

Refinement on *F*^2^	Primary atom site location: structure-invariant direct methods
Least-squares matrix: full	Secondary atom site location: difference Fourier map
*R*[*F*^2^ > 2σ(*F*^2^)] = 0.043	Hydrogen site location: inferred from neighbouring sites
*wR*(*F*^2^) = 0.135	H atoms treated by a mixture of independent and constrained refinement
*S* = 1.05	*w* = 1/[σ^2^(*F*_o_^2^) + (0.0746*P*)^2^ + 0.4733*P*] where *P* = (*F*_o_^2^ + 2*F*_c_^2^)/3
2773 reflections	(Δ/σ)_max_ < 0.001
146 parameters	Δρ_max_ = 0.37 e Å^−^^3^
0 restraints	Δρ_min_ = −0.27 e Å^−^^3^

### Special details

**Table:**

Geometry. All esds (except the esd in the dihedral angle between two l.s. planes) are estimated using the full covariance matrix. The cell esds are taken into account individually in the estimation of esds in distances, angles and torsion angles; correlations between esds in cell parameters are only used when they are defined by crystal symmetry. An approximate (isotropic) treatment of cell esds is used for estimating esds involving l.s. planes.
Refinement. Refinement of F^2^ against ALL reflections. The weighted R-factor wR and goodness of fit S are based on F^2^, conventional R-factors R are based on F, with F set to zero for negative F^2^. The threshold expression of F^2^ > 2sigma(F^2^) is used only for calculating R-factors(gt) etc. and is not relevant to the choice of reflections for refinement. R-factors based on F^2^ are statistically about twice as large as those based on F, and R- factors based on ALL data will be even larger.

### Fractional atomic coordinates and isotropic or equivalent isotropic displacement parameters (Å^2^)

**Table:**

	*x*	*y*	*z*	*U*_iso_*/*U*_eq_	
H12A	0.1707 (12)	1.015 (3)	0.2850 (10)	0.053 (5)*	
H12B	0.1337 (11)	0.802 (3)	0.2512 (9)	0.043 (4)*	
H13A	0.3357 (16)	1.149 (3)	0.4112 (13)	0.090 (7)*	
H13B	0.2620 (13)	1.136 (3)	0.4466 (12)	0.073 (6)*	
H10A	0.1961 (13)	0.509 (3)	0.3165 (11)	0.055 (5)*	
H10B	0.2701 (11)	0.531 (2)	0.3943 (10)	0.050 (4)*	
H11	0.3229 (11)	0.826 (2)	0.4490 (9)	0.047 (4)*	
C1	0.54873 (7)	0.08831 (15)	0.67511 (6)	0.0289 (2)	
C2	0.47896 (7)	0.20438 (15)	0.61002 (6)	0.0306 (2)	
H2A	0.4158	0.1689	0.6083	0.037*	
H2B	0.4853	0.1628	0.5607	0.037*	
C3	0.49064 (7)	0.43218 (15)	0.61797 (6)	0.0281 (2)	
H3A	0.5542	0.4676	0.6209	0.034*	
H3B	0.4826	0.4744	0.6666	0.034*	
C4	0.42163 (7)	0.54741 (15)	0.55112 (6)	0.0291 (2)	
C5	0.22849 (7)	0.78408 (16)	0.34760 (6)	0.0282 (2)	
N10	0.23172 (8)	0.58414 (15)	0.35140 (6)	0.0381 (3)	
N11	0.28683 (7)	0.88965 (14)	0.40661 (6)	0.0368 (2)	
N12	0.16935 (7)	0.87957 (16)	0.28669 (6)	0.0379 (3)	
N13	0.27963 (9)	1.10237 (16)	0.40542 (7)	0.0446 (3)	
O6	0.54679 (6)	−0.10939 (12)	0.66765 (5)	0.0436 (2)	
H6	0.5057	−0.1414	0.6265	0.065*	
O7	0.60494 (6)	0.17027 (12)	0.73220 (5)	0.0386 (2)	
O8	0.42768 (6)	0.74200 (11)	0.55212 (5)	0.0395 (2)	
O9	0.36190 (7)	0.45581 (13)	0.49819 (5)	0.0489 (3)	

### Atomic displacement parameters (Å^2^)

**Table:**

	*U*^11^	*U*^22^	*U*^33^	*U*^12^	*U*^13^	*U*^23^
C1	0.0314 (5)	0.0199 (4)	0.0270 (5)	−0.0002 (4)	−0.0011 (4)	0.0023 (3)
C2	0.0343 (5)	0.0190 (4)	0.0269 (5)	0.0002 (4)	−0.0052 (4)	0.0028 (3)
C3	0.0326 (5)	0.0185 (4)	0.0239 (4)	0.0009 (3)	−0.0028 (4)	0.0012 (3)
C4	0.0355 (5)	0.0195 (4)	0.0239 (4)	0.0026 (4)	−0.0012 (4)	0.0013 (3)
C5	0.0296 (5)	0.0241 (4)	0.0242 (4)	0.0005 (4)	0.0005 (4)	−0.0018 (3)
N10	0.0454 (6)	0.0221 (4)	0.0325 (5)	0.0001 (4)	−0.0058 (4)	−0.0020 (4)
N11	0.0443 (5)	0.0223 (4)	0.0285 (4)	0.0002 (4)	−0.0076 (4)	−0.0024 (3)
N12	0.0426 (5)	0.0261 (5)	0.0294 (5)	0.0009 (4)	−0.0082 (4)	0.0004 (4)
N13	0.0525 (7)	0.0224 (4)	0.0443 (6)	−0.0012 (4)	−0.0030 (5)	−0.0061 (4)
O6	0.0503 (5)	0.0181 (3)	0.0396 (5)	0.0004 (3)	−0.0147 (4)	0.0026 (3)
O7	0.0420 (5)	0.0250 (4)	0.0317 (4)	−0.0003 (3)	−0.0097 (3)	0.0007 (3)
O8	0.0485 (5)	0.0181 (3)	0.0347 (4)	0.0009 (3)	−0.0085 (3)	0.0027 (3)
O9	0.0592 (6)	0.0258 (4)	0.0348 (4)	0.0000 (4)	−0.0195 (4)	−0.0018 (3)

### Geometric parameters (Å, °)

**Table:**

C1—O7	1.2200 (13)	C5—N12	1.3207 (13)
C1—O6	1.3043 (13)	C5—N11	1.3285 (13)
C1—C2	1.4978 (13)	N10—H10A	0.840 (19)
C2—C3	1.5071 (15)	N10—H10B	0.871 (17)
C2—H2A	0.9700	N11—N13	1.4003 (15)
C2—H2B	0.9700	N11—H11	0.884 (17)
C3—C4	1.5080 (13)	N12—H12A	0.891 (19)
C3—H3A	0.9700	N12—H12B	0.852 (16)
C3—H3B	0.9700	N13—H13A	0.87 (2)
C4—O9	1.2293 (13)	N13—H13B	0.90 (2)
C4—O8	1.2804 (12)	O6—H6	0.8200
C5—N10	1.3144 (15)		
			
O7—C1—O6	120.79 (9)	O8—C4—C3	117.56 (9)
O7—C1—C2	123.16 (9)	N10—C5—N12	121.37 (10)
O6—C1—C2	116.06 (8)	N10—C5—N11	118.42 (10)
C1—C2—C3	113.65 (8)	N12—C5—N11	120.21 (10)
C1—C2—H2A	108.8	C5—N10—H10A	123.2 (12)
C3—C2—H2A	108.8	C5—N10—H10B	116.7 (11)
C1—C2—H2B	108.8	H10A—N10—H10B	119.9 (16)
C3—C2—H2B	108.8	C5—N11—N13	118.76 (9)
H2A—C2—H2B	107.7	C5—N11—H11	120.0 (10)
C2—C3—C4	113.22 (8)	N13—N11—H11	120.6 (10)
C2—C3—H3A	108.9	C5—N12—H12A	119.1 (11)
C4—C3—H3A	108.9	C5—N12—H12B	115.2 (11)
C2—C3—H3B	108.9	H12A—N12—H12B	125.7 (16)
C4—C3—H3B	108.9	N11—N13—H13A	106.3 (15)
H3A—C3—H3B	107.7	N11—N13—H13B	106.1 (13)
O9—C4—O8	121.94 (9)	H13A—N13—H13B	111 (2)
O9—C4—C3	120.50 (9)	C1—O6—H6	109.5
			
O7—C1—C2—C3	5.34 (16)	C2—C3—C4—O8	−178.55 (10)
O6—C1—C2—C3	−174.44 (10)	N10—C5—N11—N13	−175.87 (12)
C1—C2—C3—C4	178.57 (9)	N12—C5—N11—N13	4.49 (17)
C2—C3—C4—O9	1.74 (16)		

### Hydrogen-bond geometry (Å, °)

**Table:**

*D*—H···*A*	*D*—H	H···*A*	*D*···*A*	*D*—H···*A*
N10—H10B···O9	0.87 (2)	1.99 (2)	2.851 (1)	171 (2)
N11—H11···O8	0.88 (2)	2.07 (2)	2.939 (1)	166 (1)
N12—H12B···O6^i^	0.85 (2)	2.07 (2)	2.921 (1)	178 (2)
N10—H10A···O7^i^	0.84 (2)	2.05 (2)	2.886 (1)	178 (2)
O6—H6···O8^ii^	0.82	1.65	2.456 (1)	167

Symmetry codes:  (i) *x*−1/2, −*y*+1/2, *z*−1/2; (ii) *x*, *y*−1, *z*.
